# Rapid detection of avian influenza virus based on CRISPR-Cas12a

**DOI:** 10.1186/s12985-023-02232-7

**Published:** 2023-11-13

**Authors:** Xu Zhou, Siwen Wang, Yue Ma, Yanbing Li, Guohua Deng, Jianzhong Shi, Xiurong Wang

**Affiliations:** State Key Laboratory for Animal Disease Control and Prevention, Harbin Veterinary Research Institute, Chinese Academy of Agricultural Sciences, Harbin, China

**Keywords:** Avian influenza virus, CRISPR/Cas system, RT-RPA, Detection

## Abstract

**Background:**

Avian influenza (AI) is a disease caused by the avian influenza virus (AIV). These viruses spread naturally among wild aquatic birds worldwide and infect domestic poultry, other birds, and other animal species. Currently, real-time reverse transcription polymerase chain reaction (rRT-PCR) is mainly used to detect the presence of pathogens and has good sensitivity and specificity. However, the diagnosis requires sophisticated instruments under laboratory conditions, which significantly limits point-of-care testing (POCT). Rapid, reliable, non-lab-equipment-reliant, sensitive, and specific diagnostic tests are urgently needed for rapid clinical detection and diagnosis. Our study aimed to develop a reverse transcription recombinase polymerase amplification (RT-RPA)/CRISPR method which improves on these limitations.

**Methods:**

The Cas12a protein was purified by affinity chromatography with Ni-agarose resin and observed using sodium dodecyl sulfate–polyacrylamide gel electrophoresis (SDS-PAGE). Specific CRISPR RNA (crRNA) and primers targeting the M and NP genes of the AIV were designed and screened. By combining RT-RPA with the Cas12a/crRNA trans-cleavage system, a detection system that uses fluorescence readouts under blue light or lateral flow strips was established. Sensitivity assays were performed using a tenfold dilution series of plasmids and RNA of the M and NP genes as templates. The specificity of this method was determined using H1–H16 subtype AIVs and other avian pathogens, such as newcastle disease virus (NDV), infectious bursal disease virus (IBDV), and infectious bronchitis virus (IBV).

**Results:**

The results showed that the method was able to detect AIV and that the detection limit can reach 6.7 copies/μL and 12 copies/μL for the M and NP gene, respectively. In addition, this assay showed no cross-reactivity with other avian-derived RNA viruses such as NDV, IBDV, and IBV. Moreover, the detection system presented 97.5% consistency and agreement with rRT-PCR and virus isolation for detecting samples from poultry. This portable and accurate method has great potential for AIV detection in the field.

**Conclusion:**

An RT-RPA/CRISPR method was developed for rapid, sensitive detection of AIV. The new system presents a good potential as an accurate, user-friendly, and inexpensive platform for point-of-care testing applications.

## Background

Avian influenza (AI) is a syndrome caused by avian influenza viruses (AIVs) that infect poultry and wild birds. According to different clinical symptoms, AI can be divided into low-pathogenic avian influenza (LPAI) and highly pathogenic avian influenza (HPAI). AIV is an enveloped, segmented, single-stranded, negative-sense RNA virus belonging to the family *Orthomyxoviridae* and the genus *Influenza virus A* [1, 2]. According to the surface glycoproteins, hemagglutinin (HA) and neuraminidase (NA), AIVs can be classified into 16 HA and 9 NA subtypes [3, 4]. Perroncito first reported HPAI or fowl plague in 1878; since then, there have been several outbreaks worldwide, posing a great threat to the breeding industry and human health [5]. Since 2005, more than 25,000 HPAI outbreaks have occurred worldwide (https://www.woah.org/en/). Wild birds, particularly waterfowl, are natural reservoirs of all AIV subtypes, making it difficult to control the transmission of AIV [6].

Currently, the main methods commonly used for AIV detection include hemagglutination assay, hemagglutination inhibition [7], reverse transcription polymerase chain reaction (RT-PCR), and enzyme-linked immunosorbent assay (ELISA) [8]. Existing nucleic acid detection methods, such as rRT-PCR, have good sensitivity and specificity but require expensive laboratory instruments and well-trained personnel, which greatly hinders the application of this method for point-of-care testing (POCT).

**Fig. 1:**
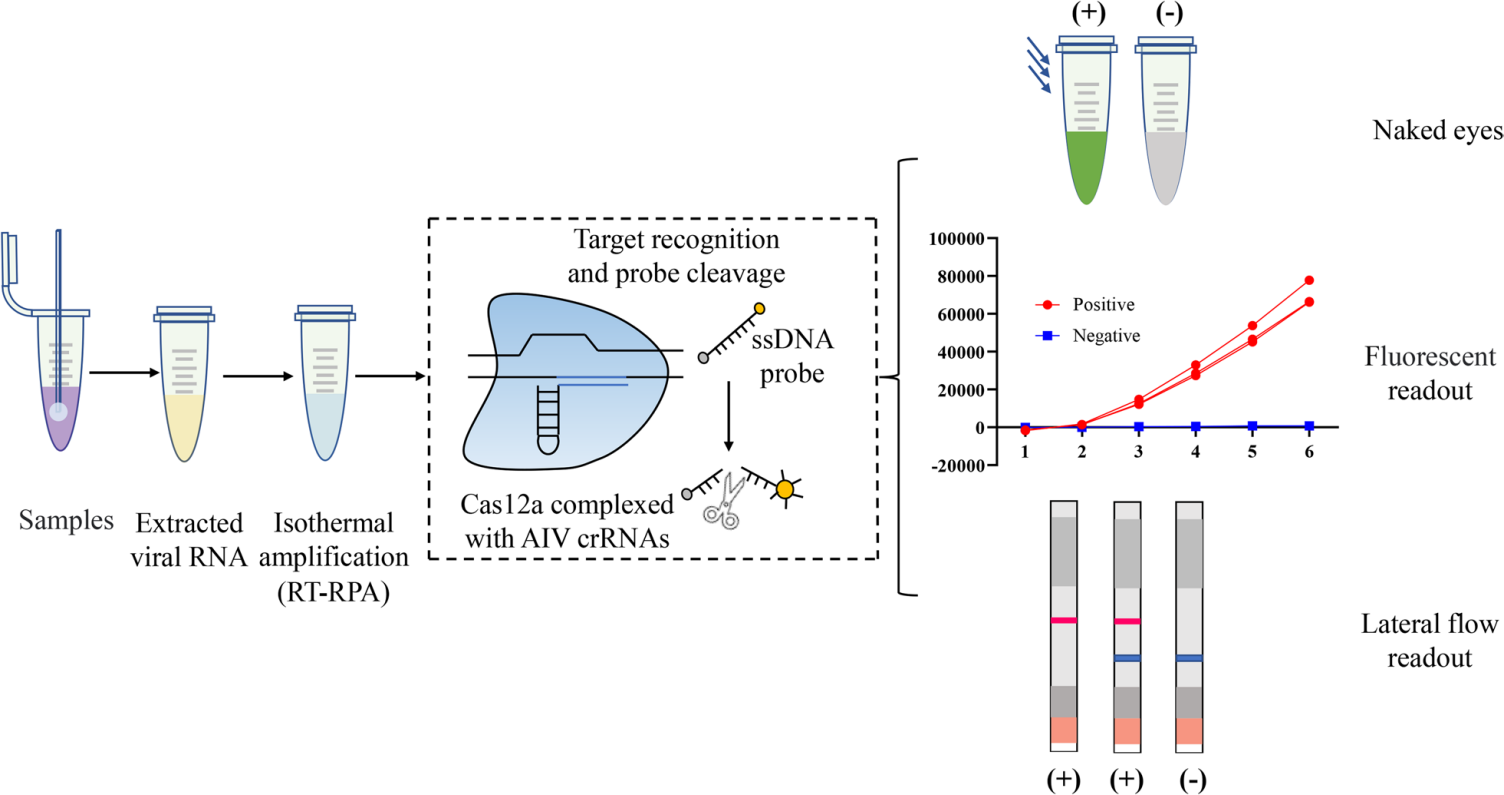
Schematic diagram of the RT/RPA assay coupled with the CRISPR-mediated detection platform for identification of AIV. Target genes are specifically amplified by RT-RPA from genomic fragments isolated from clinical samples. A specific crRNA is designed to recognize the target gene and form a complex with Cas12a and the probe. Once the target DNA is recognized by the Cas12a and crRNA complex, Cas12a exerts its non-specific endonuclease activity and cleaves the single-stranded DNA probe. By introducing a ssDNA probe that labels the fluorophore and quencher or fluorophore and biotin, the cleavage could be observed using a fluorophore reader or lateral flow dipstick

Recently, clustered regularly interspaced short palindromic repeat sequences and associated nuclease (CRISPR-Cas) systems have provided potential applications for rapid and sensitive molecular diagnostics [9–11]. The CRISPR-Cas system is an RNA-guided adaptive immune system that protects bacteria and archaea from invasion by foreign nucleic acids [12–14]. Some Cas nucleases, such as Cas12a, Cas12b, Cas13a, Cas13b, and Cas14, exert their collateral cleavage activity after recognizing specific target sequences for nucleic acid detection for diagnostic purposes [15, 16]. For example, when the Cas12a nuclease recognizes a T-rich protospacer-adjacent motif and cleaves double-stranded DNA (dsDNA) specifically at the target site guided by sequence-specific crRNA, the activated Cas nuclease indiscriminately cleaves nearby single-stranded DNA (ssDNA) labeled with quenched fluorescence or biotin. The results are displayed by fluorescent readers or lateral flow chromatography test strips (Fig. 1) [17, 18]. Nucleic acid detection technology based on the CRISPR-Cas system has been successfully applied to several highly pathogenic viruses, such as the zika virus, dengue virus, severe acute respiratory syndrome coronavirus 2 (SARS-CoV-2) [19], human papillomavirus, and AIV [9, 20, 21]. However, because Cas12a itself is not theoretically sensitive enough to detect lower levels of nucleic acids, the detection of CRISPR-Cas12a is usually combined with an isothermal amplification step such as RPA to improve its sensitivity [22, 23]. RPA is considered one of the nucleic acid amplification techniques used for molecular diagnostics, with the ability to amplify nucleic acids at the 37–42 °C temperature range for sensitive and rapid nucleic acid amplification [24]. Existing studies have confirmed that the Cas12a nuclease combined with the RPA method shows single-molecule sensitivity in a given reaction [9, 15].

In this study, a visual detection method using CRISPR-Cas12a for rapid nucleic acid detection of AIV was developed. After sample processing, by combining RT-RPA technology with the CRISPR-Cas12a system for detection, the sample can be presented in 1 h by a fluorescence reader or the naked eye. Because no sophisticated laboratory instrumentation is required, this method is useful for POCT of AIV and helps control the spread of the disease.

## Materials and methods

### Materials

*Escherichia coli* strains expressing the LbCas12a protein, plasmids containing the AIV M and NP genes, and H1–H16 subtypes of AIV, newcastle disease virus (NDV), infectious bursal disease virus (IBDV), and infectious bronchitis virus (IBV) were conserved in the State Key Laboratory of Harbin Veterinary Research Institute (HVRI), Chinese Academy of Agricultural Sciences (CAAS). A total of 81 samples were obtained from the National Reference Laboratory for Avian Influenza.

### Reagents and instruments

RNase inhibitors were obtained from Thermo Fisher Scientific. The crRNA targeting the RT-RPA of the M and NP genes was synthesized by RuiBiotech. RNA was extracted using a TIANamp Virus RNA Kit. A HiScribe T7 Quick High-Yield RNA Synthesis Kit was purchased from New England Biolabs. The single-stranded DNA reporter was synthesized by Sangon Biotech. The HybriDetect test dipstick was purchased from Warbio. The RNA Rapid Concentration Purification Kit was purchased from Sangon Biotech. Fluorescence signals were acquired using QuantStudio 5 (Applied Biosystems) and the degraded fluorescent reporter group (ssDNA-FQ) was visualized under UV light.

### Template RNA preparation

Plasmids containing the M and NP genes from AIV were PCR-amplified using primers containing T7 promoter sequences and then purified using a PCR Product Purification Kit. The purified DNA products were used as templates for in vitro transcription reactions using the HiScribe T7 Quick High Yield RNA Synthesis Kit, and RNA was purified using the RNA Rapid Concentration Purification Kit.

### Protein expression and purification

LbCas12a protein used for AIV detection was expressed in *E. coli*. The gene encoding LbCas12a was cloned into a pET-based expression vector containing a C-terminal 6 × His-tag and a TEV protease cleavage site. The soluble protein was purified as previously described with the following modifications: the bacterial expression plasmid was transformed into Rosetta^TM^2(DE3) cells, and a 10 mL bacterial culture was grown in 1 L Luria–Bertani growth media which was inoculated for growth at 37 °C and 200 RPM until an OD_600_ of 0.6. Meanwhile, LbCas12a protein expression was induced by supplementation with isopropyl β-d-1-thiogalactopyranoside to a final concentration of 0.5 mM, and the cell was cooled to 16 °C for 18 h. Bacterial cells were harvested, resuspended in lysis buffer (50 mM HEPES pH 7.2, 2 M NaCl, 20 mM imidazole, 2 mL PMSF, and 0.25 mg/mL lysozyme), disrupted by sonication, and purified using an Ni–NTA column. The purified protein was added to TEV enzyme to remove the His-tag and concentrated through an ultrafiltration tube to obtain the purified LbCas12a protein, which was stored at -80 °C or used directly in the assay.

### Optimization of RT-RPA and Cas12a detection

RNase H is an endonuclease that specifically hydrolyzes RNA strands in RNA-cDNA hybrids. In RT-RPA, viral RNA is reverse transcribed into cDNA or cDNA-RNA hybrids using reverse transcriptase, which helps to improve amplification efficiency [25]. To obtain the best amplification, different concentrations of RNase H were added to the RT-RPA reaction, and the amplification products were detected for Cas12a by reading the fluorescence signal to determine the optimal concentration.

To establish the optimal reaction conditions for Cas12a detection, the influence of different factors, including pH, ssDNA reporter, buffer composition, Mg^2+^, and crRNA concentration, on Cas12a cleavage efficiency was investigated.

### Design and screening for crRNA and primers of RT-RPA

Based on the conserved sequences of the M and NP genes of AIV, four M-crRNAs and four NP-crRNAs were designed and screened for the best crRNA. Based on the selected crRNA, RT-RPA primers targeting the M and NP genes of AIV were designed as described for Twist-Dx (Maidenhead, United Kingdom). To screen the best pairs, a random combination of primers was used to screen the primer pairs with the most efficient amplification effects, as shown in Table 1. Primer pairs with the most efficient performance were used in subsequent experiments.

**Table 1:** Oligonucleotides used in this experiment

Primer name	Sequence (5′–3′)	Product size (bp)
crRNA-M-1	UAAUUUCUACUAAGUGUAGAUAAGAAAAGACGAUCAAGAAUCC	
crRNA-M-2	UAAUUUCUACUAAGUGUAGAUCAGGCCUACCAGAAACGGAU	
crRNA-M-3	UAAUUUCUACUAAGUGUAGAUCACUCCCATCCGUUUCUGG	
crRNA-M-4	UAAUUUCUACUAAGUGUAGAUUGUUCACGCUCACCGUGCCCAG	
mRPA-4-F1	CAAGACCAATC CTGTCACCTCTGACTAAGGG G	103
mRPA-4-R1	TTTTGGACAAAGCGTCTACGCTGCAGTCCTCGCTC	
mRPA-4-F2	CAATC CTGTCACCTCTGACTAAGGGGATTTTAGGG	97
mRPA-4-R2	TTTTGGACAAAGCGTCTACGCTGCAGTCCTCGCTC	
mRPA-4-F3	AGATCG CGCAGAGACTTGAGGATGTCTTTGCAGGG	214
mRPA-4-R3	TCCATGTTGTTTGGGTCTCCATTTCCATTTAGGGC	
crRNA-NP-1	UAAUUUCUACUAAGUGUAGAUCGUCUGCUUCAAAACAGCCAG	
NP1-RPA-F1	TGGATATGACTTTGAGAGAGAAGG GTACTCCCTGG	137
NP1-RPA-R1	ATGCCATCCACACTAGTTGACTCTTGTGTGCTGGG	
NP1-RPA-F2	TATGACTTTGAGAGAGAAGGGTACTCCCTGGTTGG	212
NP1-RPA-R2	GATAGCTGTCCTCTTGGGACCATTCTTGTCCCTC	
NP1-RPA-F3	GAGAGAGAAGGGTACTCCCTGGTTGGAATAGATCC	89
NP1-RPA-F3	TTCTCATTTGGTCTAATGAGACTAAAGACCTGGC	
crRNA-NP-2	UAAUUUCUACUAAGUGUAGAUCCGGAGAAGAGACGGGAAAUGG	
crRNA-NP-3	UAAUUUCUACUAAGUGUAGAUUGGCAAGGUCUGCACUCAUCCU	
crRNA-NP-4	UAAUUUCUACUAAGUGUAGAUGAAUUUCCCUUUGAGGAUGUUGC	

### RT-RPA reaction

RT-RPA reactions were performed using a RT-Basic RNA isothermal rapid amplification kit (Genenode, Wuhan, China) according to the manufacturer’s protocol. In brief, the reactions were performed at a total volume of 50 μL comprising RT-RPA enzymes, 2 μL RNA input, 29.4 μL A Buffer, 2 μL Forward primer (10 μM), 2 μL Reverse primer (10 μM), and 2.5 μL B Buffer. All reactions were incubated at 42 °C for 30 min in a PCR instrument or metal bath.

### Cas12a detection reactions

Detection assays were performed with 10 μL RT-RPA products or 1 μL dsDNA plasmid, 1.25 μL purified Cas12a protein, 12.5 μL reaction Buffer (20 mM Tris–HCl pH 7.5, 100 mM KCl, 5 mM MgCl_2_, 1 mM DTT, 5% glycerol), 1 μL crRNA (200 nM), 0.25 μL RNase inhibitor (RI), 1 μL ssDNA reporter, and water to make up a total volume of 25 μL. Reactions were incubated in a Real-Time PCR Detection System (ABI QuantStudio 5) or a fluorescence plate reader (Enspire, USA) for 1–2 h at 37 °C with fluorescence signals measured every 5 min.

### Lateral flow detection

After amplification was completed, 2 μL of amplification product was mixed with 25 μL reaction buffer, 2.5 μL LbCas12a, 2 μL crRNA, 2 μL probe (FAM-TTATT-Biotin), 0.5 μL RI, and 16 μL ddH_2_O, and given at 37 °C for 2 h. A side-flow test strip was then added to the reaction tube and the results were observed after 2 min. A single band near the sample pad indicated a negative result, whereas a single band or two bands near the top of the test strip indicated a positive result.

### rRT-PCR assay for AIV testing

rRT-PCR detection of the AIV-M gene was performed using the QuantStudio 5 system according to the manufacturer’s instructions (Guanmu Biotechnology, Hunan, China). In brief, single-tube PCRs were prepared including 19 μL reaction buffer, 1 μL enzyme mixture, and 5 μL RNA template. The amplification program was reverse transcription at 50 °C for 2 min, pre-denaturation at 95 °C for 2 min, followed by 40 cycles of denaturation at 95 °C for 15 s and annealing and extension at 60 °C for 30 s. Fluorescence signals were collected during the annealing and extension steps per cycle.

### Statistical analysis

The data were analyzed using GraphPad Prism 8.0 (GraphPad Software, Inc.) for analysis of variance. Data are presented as the mean ± standard deviation of three independent experiments.

## Results

### Protein purification and verification of Cas12a protein activity

**Fig. 2:**
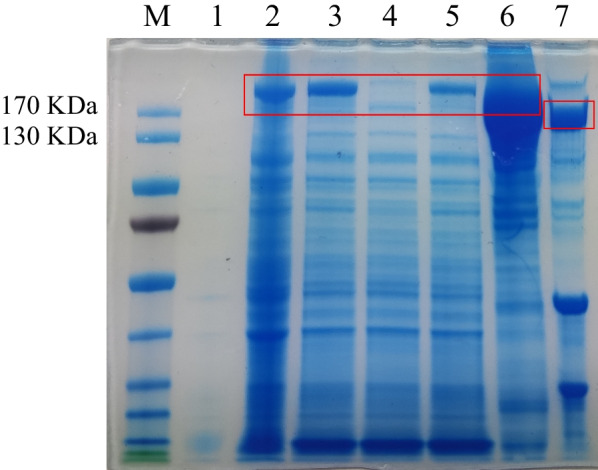
SDS-PAGE gel plots of each sample of Cas12a. A: LbCas12a protein purification graph. M: Marker; 1: bacterial sedimentation before induction; 2: bacterial sedimentation after induction; 3: supernatant; 4: flow-through solutions; 5: washing solutions; 6: eluent; 7: proteins after enzymatic digestion with TEV enzyme

Sodium dodecyl sulfate–polyacrylamide gel electrophoresis (SDS-PAGE) was performed on the bacterial pellet (before and after induction), supernatant, flow-through solutions, washing solutions, eluent, and proteins after digestion with the TEV enzyme (Fig. 2). The molecular weight of the original LbCas12a protein was 192.1 kDa. The actual protein size of LbCas12a after excision of the tag using the TEV enzyme was 143.7 kDa. According to the SDS-PAGE results, the Cas12a protein was highly expressed.

### Optimization of reaction system

**Fig. 3:**
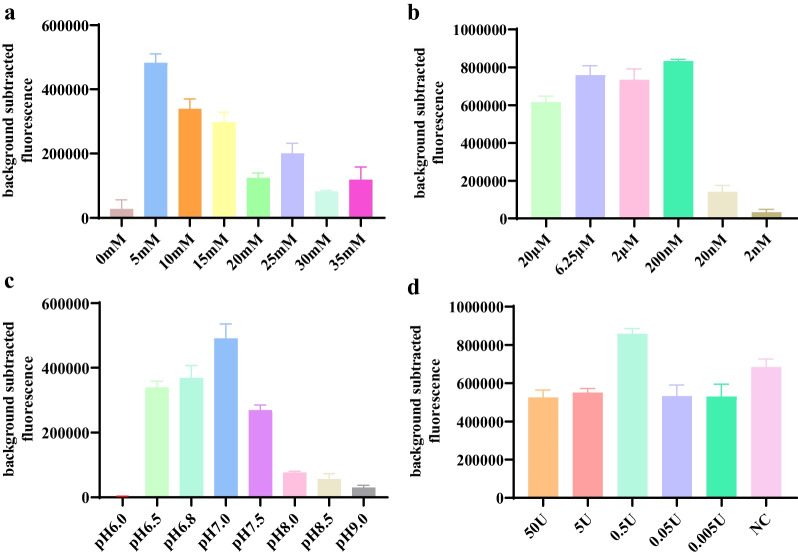
Optimization of the RT-RPA and CRISPR/Cas12a system. **a** Cleavage effect of reaction buffers with different Mg2^+^ concentrations on the CRISPR/Cas12a assay. **b** Cleavage effect of different dilution concentrations of crRNA on Cas12a protein cleavage efficiency. **c** Cleavage effect of reaction buffers with different pH values on the CRISPR/Cas12a assay. **d** Effect of RNase H nuclease on RT-RPA assays

In this experiment, the reaction system, including RNase H, crRNA concentration, Mg2^+^, pH, ssDNA reporter, and reaction buffer, was optimized to determine the optimal reaction system to fully utilize the cleavage efficiency of the Cas12a protein (Fig. 3). The results showed that the cleavage efficiency of Cas12a was highest when RNase H nuclease, 0.5 mM Mg2^+^, pH 7, and 200 nM crRNA were used.

### Screening of crRNA and primers

**Fig. 4:**
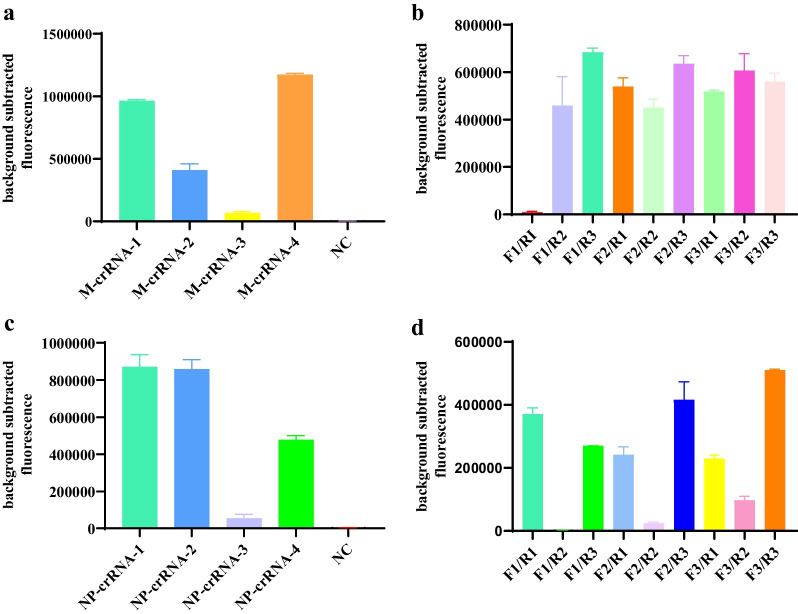
Design and screening of crRNA and RPA primers. **a** Cleavage activity of CRISPR/Cas12a induced by four crRNAs targeting the AIV M gene, **b** primer screening for the M gene, **c** cleavage activity of CRISPR/Cas12a induced by four crRNAs targeting the AIV NP gene, and **d** primer screening for the NP gene. A comprehensive screen using a random combination of primers was used to identify the primer pairs with the best performance. Fluorescence signals were collected using the QuantStudio software (Applied Biosystems)

Four crRNA sequences were identified based on the conserved sequences of the AIV M and NP genes, and the best crRNA sequences were determined using the fluorescence method. The results showed that M-crRNA-4 (Fig. 4a) and NP-crRNA-1 (Fig. 4c) had better effects, and the screened crRNAs were used in subsequent experiments.

Because the sequences of the amplification primers are critical for RPA, primers must be screened to determine the best detection. For each reaction, nine primer pairs targeting M (Fig. 4b) and NP genes (Fig. 4d) were tested using 1.17 × 10^12^ copies/μL virus RNA as templates, and the best primer pairs (F1/R3) and (F3/R3) were identified for subsequent experiments.

### Sensitivity and specificity tests

**Fig. 5:**
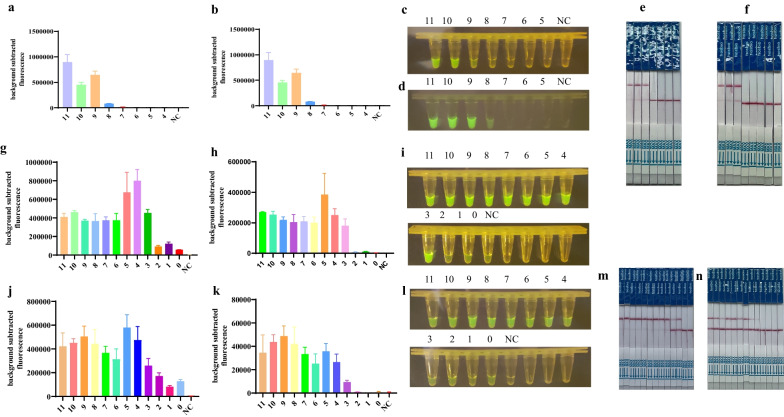
Sensitivity analysis. **a**, **c**, **e** Ten-fold serial dilutions of plasmid template targeting the M gene at 2.4 × 10^11^ copies/µl for the sensitivity assay; **b**, **d**, **f** tenfold serial dilutions of plasmid template targeting the NP gene at 8.67 × 10^11^ copies/µl for the sensitivity assay; (M:11–4, 2.4 × 10^11^ copies/µl–2.4 × 10^4^ copies/µl; NP:11–4, 8.67 × 10^11^ copies/µl–8.67 × 10^4^ copies/µl); **g**, **h**, **i**, **m** tenfold serial dilutions of RNA template targeting M gene at 6.7 × 10^11^ copies/μL for the sensitivity assay by RT-RPA/CRISPR; **i**, **k**, **l**, **n** tenfold serial dilutions of RNA template targeting NP gene at 12 × 10^11^ copies/μL for the sensitivity assay by RT-RPA/CRISPR. (M:11–0, 6.7 × 10^11^ copies/µl–6.7 × 10^0^ copies/µl; NP:11–0, 12 × 10^11^ copies/µl–12 × 10^0^ copies/µl). Fluorescent signals were collected every 5 min and displayed for 2 h and 30 min, respectively

To determine the analytical sensitivity of this system, the M and NP plasmids and RNA were used as templates for detection. The CRISPR/Cas12a system was able to detect a sensitivity of 2.4 × 10^8^ copies/μL and 8.67 × 10^8^ copies/μL using plasmids including M and NP genes as templates, respectively, at tenfold serial diluted concentrations (Fig. 5a–f). Next, tenfold serial dilutions of AIV RNA were used as templates for the detection of the M and NP genes (Fig. 5g–n). RT-RPA amplification was performed for the M and NP genes and the amplified products were examined using CRISPR/Cas12a. The results showed that after combining RT-RPA, the sensitivity of CRISPR/Cas12a detection could reach 6.7 copies/μL and 12 copies/μL.

**Fig. 6:**
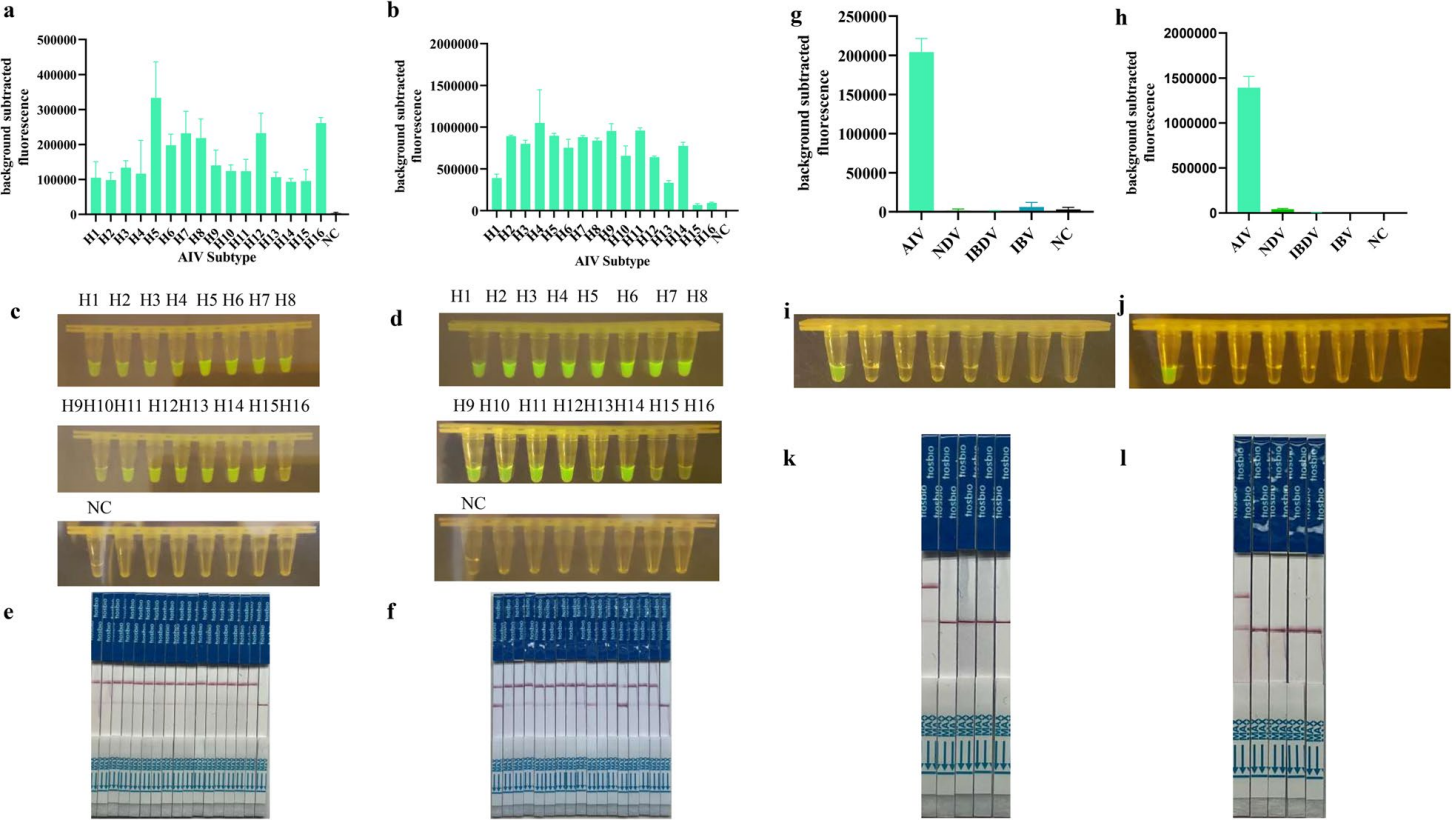
Specificity analysis. RNA of AIVs, IBDV, NDV, and IBV were used as templates for RT-RPA/CRISPR specificity; **a**, **c**, **e** the RNA of 16 HA subtypes of AIV was used as templates, the M gene was targeted for RT-RPA/CRISPR sensitivity detection; **b**, **d**, **f** the RNA of 16 HA subtypes of AIV was used as templates, the NP gene was targeted for RT-RPA/CRISPR sensitivity detection; **g**, **i**, **k** specificity assay with the M gene as target; **h**, **j**, **l** specificity assay with the NP gene as target

In this experiment, RT-RPA/CRISPR was performed using the M and NP (Fig. 6a–f) genes of the H1-H16 subtypes of AIV as templates, and the results showed that this method was able to successfully detect 16 HA subtypes of AIV and determine the reliability of the assay. In addition, to examine the specificity of RT/RPA-CRISPR for AIV detection, the established assay was used to detect other avian pathogens, including NDV, IBV, and IBDV (Fig. 6g–l). The results showed that all tested avian pathogens were negative except AIV, which was positive, indicating that this method is highly specific for the detection of AIV.

### Assessment of RT-RPA/CRISPR assays from avian clinical samples for consistency with commercial rRT-PCR kits

To assess the performance of the RT/RPA-CRISPR assay, 81 clinical samples, including swabs and lungs, were analyzed using both RT/RPA-CRISPR and a commercial rRT-PCR assay, as shown in Table 2.

**Table 2:** The performance of RT/RPA-CRISPR for AIV detection in clinical samples compared with real-time RT-PCR

Methods	CRISPR/Cas12a-RT/RPA		
rRT-PCR	Positive	Negative	Total
Positive	53	1	54
Negative	1	26	27
Total	54	27	81
Coincidence rate: 97.5%			
Sensitivity: 100%			
Specificity: 100%			

## Discussion

To date, the prevalence of AIV in most countries worldwide has caused serious socioeconomic impact, restricting the trade of poultry products, affecting food safety, and posing a great health threat to humans [26, 27]. Currently, the main molecular diagnostic test for AIV is rRT-PCR, which has the advantages of high sensitivity and short turnaround time [28], but requires expensive instruments and professional operating personnel; therefore, it is inconvenient for POCT. In this study, a simple and sensitive RT-RPA method coupled with CRISPR/Cas12a was developed for rapid AIV detection.

Notably, off-target and mismatch recognition effects could be major concerns in CRISPR/Cas12a-mediated nucleic acid tests. To solve these problems, four crRNAs each targeting the conserved sequences of the M and NP genes of AIV were designed and synthesized by screening and comparing the target sequences. Although the RPA technique has been successfully applied for the detection of many pathogens because of its high sensitivity and efficiency [29, 30], it suffers from some intrinsic drawbacks, such as the issue of detection of RPA products, design rules, result determination of primers and probes, and the current lack of software for designing RPA primers. Therefore, in this study, primers were rigorously designed to achieve better amplification of the conserved target sequence.

The ability of the RT/RPA-CRISPR method to detect AIV has been demonstrated. The CRISPR/Cas12a-mediated nucleic test fluorescence reporter system achieved a sensitivity level of 2.4 × 10^8^ copies/μL without RT-RPA. In combination with RT-RPA amplification, the RT/RPA-CRISPR test detected RNA targets at a sensitivity level of 6.7 copies/μL and 12 copies/μL targeting the M and NP genes respectively. The crude extraction method (high-temperature or lysis buffer) can be used in the field with slightly decreased sensitivity, and further research is needed to develop a more suitable and optimized extraction method to meet the needs of on-site sample detection.

Visual detection is crucial for nucleic acid diagnosis, particularly in the absence of experimental instrumentation. In this study, a reporter labeled with FAM on the 5′ end and BHQ1 on the 3′ end was used. After Cas12a protein cleavage, green signals were observed with the naked eye under UV irradiation when the target was present. With the method, a detection sensitivity of 6.7 copies/μL was achieved, which is comparable with the fluorescence plate reader method. In addition, another reporter labeled with FAM molecules at the 5′ end and biotin at the 3′ end was developed. When bound to lateral flow strips, the disruption of the reporter was visible to the naked eye. This method exhibited slightly lower analytical sensitivity to the template than the fluorometric method because of the influence of the strip itself [31].

Next, the sensitivity of the RT-PCR/CRISPR assay was compared with that of the rRT-PCR and RT/RPA-only methods. These results indicate that RT-RPA/CRISPR can be detected with a somewhat higher sensitivity than real-time fluorescent RT-PCR and RT-RPA-only detection methods, an observation that may be attributed to the signal amplification effect induced by the trans-cleavage activity of Cas12a.

In summary, an RT-RPA/CRISPR-Cas12a method was developed for the rapid and sensitive detection of AIV. By combining the lateral flow strips and RT-RPA/CRISPR-Cas12a, this detection system has several advantages, including high sensitivity, low reliance on sophisticated instruments and trained specialists, short assay time, ease of operation, cost-effectiveness, and enhanced testing accuracy [32]. The new system has strong potential as an accurate, user-friendly, and inexpensive platform for point-of-care testing applications in CRISPR-based diagnostics.

## Data Availability

All data generated and analyzed during this study are included in this published article.

## Abbreviations

AI: Avian influenza
AIV: Avian influenza virus
POCT: Point-of-care testing
SDS-PAGE: Sodium dodecyl sulfate–polyacrylamide gel electrophoresis
rRT-PCR: Real-time reverse transcription-polymerase chain reaction
RT-PPA: Reverse transcription-recombinase polymerase amplification
CRISPR-Cas: Clustered regularly interspaced short palindromic repeats and associated protein
LPAI: Low pathogenic avian influenza
HPAI: Highly pathogenic avian influenza
HI: Hemagglutination inhibition assay
ELISA: Enzyme-linked immunosorbent assay
dsDNA: Double-stranded DNA
ssDNA: Single-stranded DNA
SARS-CoV-2: Severe Acute Respiratory Syndrome Coronavirus 2
NDV: Newcastle Disease Virus
IBV: Infectious Bronchitis Virus
IBDV: Infectious Bursal Disease Virus
HVRI: Harbin Veterinary Research Institute
CAAS: Chinese Academy of Agricultural Sciences
