# Correction: Rapid detection of avian influenza virus based on CRISPR-Cas12a

**DOI:** 10.1186/s12985-024-02436-5

**Published:** 2024-08-02

**Authors:** Xu Zhou, Siwen Wang, Yue Ma, Yanbing Li, Guohua Deng, Jianzhong Shi, Xiurong Wang

**Affiliations:** State Key Laboratory for Animal Disease Control and Prevention, Harbin Veterinary Research Institute, Chinese Academy of Agricultural Sciences, Harbin, China

**Correction: Virol J 20, 261 (2023)**

**https://doi.org/10.1186/s12985-023-02232-7**

Following publication of the original article [1], the authors identified some errors in Fig. 5 (a b e) and Table 1 (line 18). The correct Fig. 5 and Table 1 is given below:

**Fig. 5:**
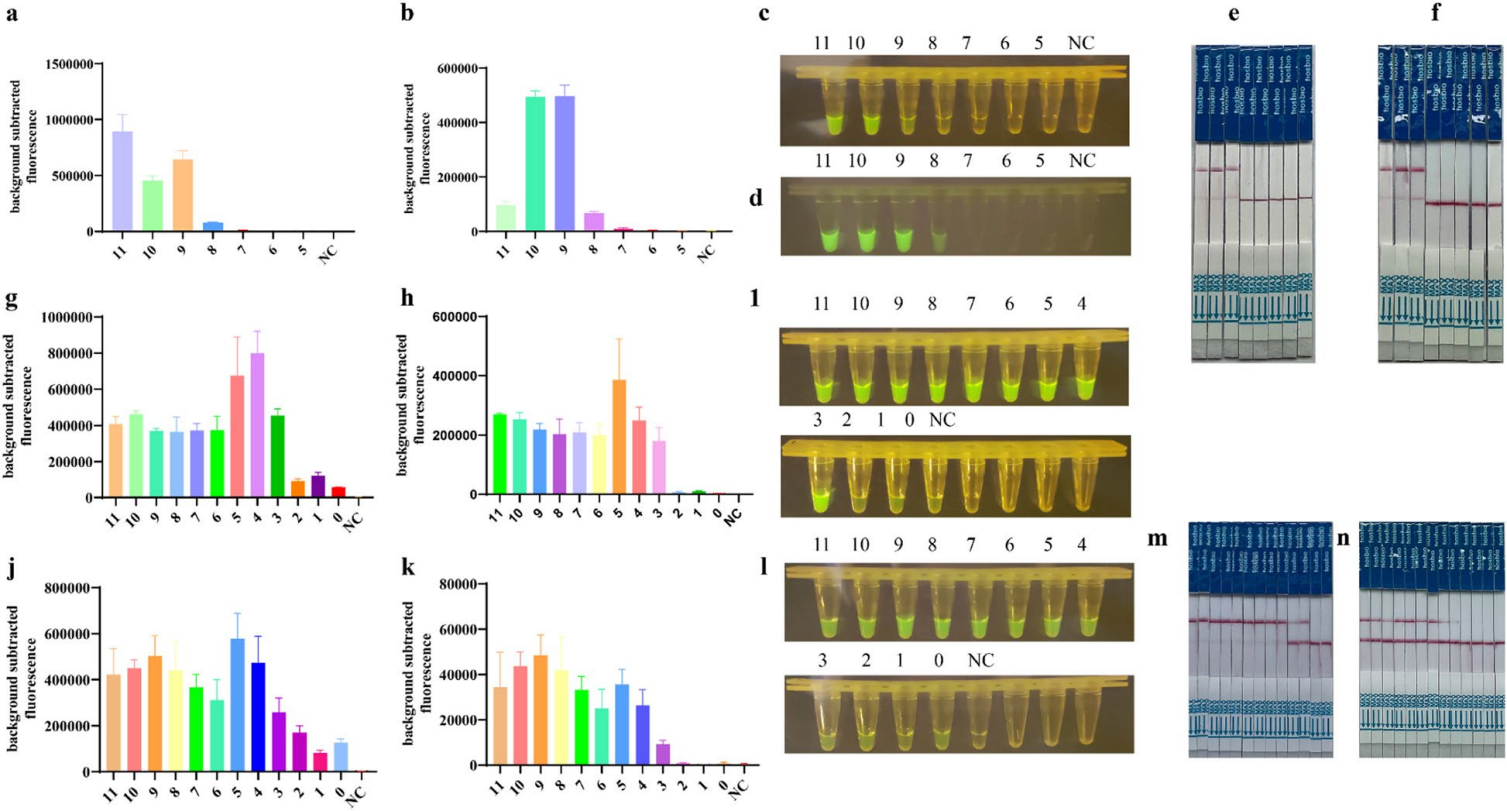
Sensitivity analysis. a, c, e Ten-fold serial dilutions of plasmid template targeting the M gene at 2.4 × 10^11^ copies/µl for the sensitivity assay; b, d, f Ten-fold serial dilutions of plasmid template targeting the NP gene at 8.67 × 10^11^ copies/µl for the sensitivity assay; (M:11–5, 2.4 × 10^11^ copies/µl-2.4 × 10^5^ copies/µl; NP:11–5, 8.67 × 10^11^ copies/µl-8.67 × 10^5^ copies/µl); g, h, i, m Ten-fold serial dilutions of RNA template targeting M gene at 6.7 × 10^11^ copies/μL for the sensitivity assay by RT-RPA/CRISPR; i, k, l, n Ten-fold serial dilutions of RNA template targeting NP gene at 12 × 10^11^ copies/μL for the sensitivity assay by RT-RPA/CRISPR. (M:11–0, 6.7 × 10^11^ copies/µl-6.7 × 10^0^ copies/µl; NP:11–0, 12 × 10^11^ copies/µl-12 × 10^0^ copies/µl). Fluorescent signals were collected every 5 min and displayed for 2 h and 30 min, respectively

**Table 1:** Oligonucleotides used for crRNA and RT-RPA production

Primer name	Sequence(5'to3')	Product size
crRNA-M-1	UAAUUUCUACUAAGUGUAGAUAAGAAAAGACGAUCAAGAAUCC	
crRNA-M-2	UAAUUUCUACUAAGUGUAGAUCAGGCCUACCAGAAACGGAU	
crRNA-M-3	UAAUUUCUACUAAGUGUAGAUCACUCCCATCCGUUUCUGG	
crRNA-M-4	UAAUUUCUACUAAGUGUAGAUUGUUCACGCUCACCGUGCCCAG	
mRPA-4-F1	CAAGACCAATCCTGTCACCTCTGACTAAGGGG	103 bp
mRPA-4-R1	TTTTGGACAAAGCGTCTACGCTGCAGTCCTCGCTC	
mRPA-4-F2	CAATCCTGTCACCTCTGACTAAGGGGATTTTAGGG	97 bp
mRPA-4-R2	TTTTGGACAAAGCGTCTACGCTGCAGTCCTCGCTC	
mRPA-4-F3	AGATCGCGCAGAGACTTGAGGATGTCTTTGCAGGG	214 bp
mRPA-4-R3	TCCATGTTGTTTGGGTCTCCATTTCCATTTAGGGC	
crRNA-NP-1	UAAUUUCUACUAAGUGUAGAUCGUCUGCUUCAAAACAGCCAG	
NP1-RPA-F1	TGGATATGACTTTGAGAGAGAAGGGTACTCCCTGG	137 bp
NP1-RPA-R1	ATGCCATCCACACTAGTTGACTCTTGTGTGCTGGG	
NP1-RPA-F2	TATGACTTTGAGAGAGAAGGGTACTCCCTGGTTGG	212 bp
NP1-RPA-R2	GATAGCTGTCCTCTTGGGACCATTCTTGTCCCTC	
NP1-RPA-F3	GAGAGAGAAGGGTACTCCCTGGTTGGAATAGATCC	89 bp
NP1-RPA-R3	TTCTCATTTGGTCTAATGAGACTAAAGACCTGGC	
crRNA-NP-2	UAAUUUCUACUAAGUGUAGAUCCGGAGAAGAGACGGGAAAUGG	
crRNA-NP-3	UAAUUUCUACUAAGUGUAGAUUGGCAAGGUCUGCACUCAUCCU	
crRNA-NP-4	UAAUUUCUACUAAGUGUAGAUGAAUUUCCCUUUGAGGAUGUUGC	
